# Lights Off for Arbuscular Mycorrhiza: On Its Symbiotic Functioning under Light Deprivation

**DOI:** 10.3389/fpls.2016.00782

**Published:** 2016-06-06

**Authors:** Tereza Konvalinková, Jan Jansa

**Affiliations:** ^1^Laboratory of Fungal Biology, Institute of Microbiology, The Czech Academy of SciencesPrague, Czech Republic; ^2^Department of Experimental Plant Biology, Faculty of Science, Charles University in PraguePrague, Czech Republic

**Keywords:** mycorrhizal symbiosis, costs and benefits, light intensity, shading duration, plant growth, phosphorus acquisition, common mycorrhizal networks

## Abstract

Plants are often exposed to shade over different time scales and this may substantially affect not only their own growth, but also development and functioning of the energetically dependent organisms. Among those, the root symbionts such as arbuscular mycorrhizal (AM) fungi and rhizobia represent particularly important cases—on the one hand, they consume a significant share of plant carbon (C) budget and, on the other, they generate a number of important nutritional feedbacks on their plant hosts, often resulting in a net positive effect on their host growth and/or fitness. Here we discuss our previous results comparing mycorrhizal performance under different intensities and durations of shade (Konvalinková et al., 2015) in a broader context of previously published literature. Additionally, we review publicly available knowledge on the root colonization and mycorrhizal growth responses in AM plants under light deprivation. Experimental evidence shows that sudden and intensive decrease of light availability to a mycorrhizal plant triggers rapid deactivation of phosphorus transfer from the AM fungus to the plant already within a few days, implying active and rapid response of the AM fungus to the energetic status of its plant host. When AM plants are exposed to intensive shading on longer time scales (weeks to months), positive mycorrhizal growth responses (MGR) are often decreasing and may eventually become negative. This is most likely due to the high C cost of the symbiosis relative to the C availability, and failure of plants to fully compensate for the fungal C demand under low light. Root colonization by AM fungi often declines under low light intensities, although the active role of plants in regulating the extent of root colonization has not yet been unequivocally demonstrated. Quantitative information on the rates and dynamics of C transfer from the plant to the fungus is mostly missing, as is the knowledge on the involved molecular mechanisms. Therefore, these subjects deserve particular attention in the future.

## Introduction

**Arbuscular mycorrhizal (AM) symbiosis** is a widespread natural phenomenon, involved in mineral nutrition of a great majority of the terrestrial plant species, and in carbon (C) cycling between the plants and soil (Smith and Read, 2008; Drigo et al., 2010). This relationship is unspecific, with many host plant species (at least potentially) being colonized by the same fungal symbiont (van der Heijden et al., 2008). Due to its widespread nature and the fact that many important agricultural crops (e.g., wheat, rice, soybean, maize, potato etc.) establish this kind of **symbiosis**, it has been intensively studied over several decades mainly from the point of **symbiotic benefits** provided to its host plant. Among those, improved acquisition of phosphorus (P) by mycorrhizal plants as compared to their non-mycorrhizal counterparts is considered to be most important. This is because P is often the limiting resource for plant growth in a lot of natural as well as agricultural habitats; it has a low mobility in soils and the AM fungal hyphae, extending up to several cm from the roots, markedly increase access for plants to this soil resource (Jakobsen et al., 1992; Jansa et al., 2003, 2005; Cardoso et al., 2006; Jemo et al., 2014). The AM fungi are supposed to have better access to the sparsely distributed soil sources as compared to the plant roots (Smith and Read, 2008; Neumann and George, 2010) due to their very thin (3–7 μm) hyphae, which can explore wider area with lower overall expense (be it C or energy) than the roots. AM fungi can also reach narrow soil pores physically inaccessible to roots, in which water holds and nutrients are dissolved longer than in larger soil pores (Neumann and George, 2010). The symbiosis with AM fungi confers also other nutritional and non-nutritional benefits such as improved zinc, sulfur and nitrogen (N) acquisition (Jansa et al., 2003; van der Heijden et al., 2008; Casieri et al., 2012) and improved drought and pathogen tolerance (Newsham et al., 1995; Augé et al., 2003, 2014), which may be important under specific circumstances and/or environmental contexts (Javaid, 2009). However, because the AM fungi have higher N concentrations in their tissues than the plants, they could compete with their hosts for this nutrient, especially under severe N limitation (Johnson et al., 2015).

KEY CONCEPT 1 **Symbiosis**

Persistent interaction between organisms belonging to different species, including reciprocally positive (mutualism) as well as unequal relationships (e.g., parasitism).

KEY CONCEPT 2 **Arbuscular mycorrhizal (AM) symbiosis**

Coexistence of plants with fungi from phylum Glomeromycota. The hyphae provide direct connection of the root inner cortex with the soil, supplying the plant with mineral nutrients. The fungi lack saprotrophic capacity and obtain all their carbon from the plant host. AM symbiosis is generally considered to be mutualistic, but this is not necessarily true under all circumstances.

KEY CONCEPT 3 **Symbiotic benefit**

Actual advantage, which an organism derives from the symbiotic relationship (gross benefits, e.g., plant phosphorus uptake via mycorrhizal hyphae) or the difference (measured or hypothetical) in growth/nutrition/fitness between the same organism living with and without the symbiont (net benefits).

Since the AM fungi are completely dependent on supply of photosynthetically fixed C from their hosts for their metabolism and growth (Bago et al., 2000), the C supply from the plant can be regarded as an infinitely large benefit for AM fungal fitness. On the other hand, from the plant's perspective, the amount of C provided to the fungal symbiont represents the **symbiotic costs**. Previous research has mostly shown figures between 4 and 10% of the plant photosynthetic production to be allocated to the AM fungal symbiont (Paul and Kucey, 1981; Grimoldi et al., 2006; Lendenmann et al., 2011; Calderón et al., 2012), whereas the highest reported value is 20% (Jakobsen and Rosendahl, 1990). Under sufficient light, the levels of plant photosynthesis could be upregulated to compensate for the increased C sink strength (Kaschuk et al., 2009). However, under conditions where light limits the photosynthesis, symbiotic costs could rapidly become a large part of the plant C budget, with pronounced consequences for plant C allocation, functioning and ultimately the growth. Such conditions are very common in nature and may occur regularly and predictably (e.g., night, monsoons, or variation in canopy transparency of deciduous trees) as well as erratically, extending at time scales from minutes to weeks (thunderstorms, cloudy weather, closing canopies of neighboring plants, or development of microbial biofilms on leaves). Plants have several possible ways to optimize their energy balance under shading conditions, such as adjusting morphology of the shoots (elongation) or the leaves (e.g., increasing their surface, Valladares and Niinemets, 2008) or to reduce the assimilate supply to the symbionts like mycorrhizal fungi or rhizobia. Several studies have now provided insights into the dynamics of symbiotic C allocation under changing light conditions and the consequences thereof for the rates of root colonization by AM fungi, plant nutrition, and growth (e.g., Fellbaum et al., 2012, 2014, and the references cited in Table 1).

KEY CONCEPT 4 **Symbiotic cost**

Actual losses due to a symbiotic relationship by a given partner (gross cost, e.g., carbon taken away from the plant by the mycorrhizal fungus) or the difference (measured or hypothetical) in growth/nutrition/fitness between the same organism living without and with the symbiont (net cost).

**Table 1:** **Synthesis of previously published literature on the effects of experimentally manipulated light intensities on the mycorrhizal growth response (MGR)**.

**No**.	**Plant species**	**AM species**	**PPFD (μmol m^−2^ s^−1^)**	**Shading duration (days)**	**MGR**	**Effect of light**		**References**
						**MGR**	**% col**.	
1	*Lycopersicum esculentum*	*G. intraradices*	600, 225	22	−	+	+	Marschner and Timonen, 2005
2	*Glycine max*^1^	*G. fasciculatum*	700, 350, 170	80	+	+	+	Bethlenfalvay and Pacovsky, 1983
3	*Trifolium subterraneum*^1^	*G. mosseae*	450, 100	42	+	+	0	Tester et al., 1985^a^
4	*Allium cepa*	“*Endogone”*	(430, 224)^b^	70	+	+	0	Hayman, 1974
5	*Allium cepa*	*G. mosseae*	550/600, 250	14, 28, 42, 56	+	+	0	Son and Smith, 1988^c^
6	*Allium cepa*	*G. mosseae*	410, 190	42	+	+	0	Smith and Gianinazzi-Pearson, 1990^c^
7	*Allium vineale*	*C. candidum*	1339, 662	28, 42, 56	+	+	?	Zheng et al., 2015
8	*Acmena resa*^2^	field AM fungi	157, 54	180	+	+	0	Gehring, 2003
9	*Dicorynia guianensis*^2^	field soil	[sun 50%, 14%, 1%]	350	+	+	±	Bereau et al., 2000
10	*Persea americana*^2^	*G. intraradices*	1250, 125	180	+	+	0	Violi et al., 2007
11	*Sorghum vulgare*^4^	*G. fasciculatum*	418, 308, 204	35	+	+	0/+^d^	Graham et al., 1982^c^
12	*Andropogon gerardii*^4^	field soil	618−1047, 66%, 33%	98	+	+/±^e^	+	Johnson et al., 2015^c^
13	*Allium porrum*	*G. mosseae*	515, 250	14, 28, 42, 56	+∕−^d^	(+∕−)^d^^,^ ^f^	0	Pearson et al., 1991
14	*Pisum sativum*^1^	*G. mosseae*	390, 190	35	−	−	+	Reinhard et al., 1993
15	*Trifolium subterraneum*^1^	*G. intraradices*	270, 68	14	−	0	+	Olsson et al., 2010
16	*Elymus repens*^3^	field soil	[glasshouse, 70%]	84	−	0	+	Grman, 2012
17	*Bromus inermis*^3^	field soil	[glasshouse, 70%]	84	−	0	0	Grman, 2012
18	*Schizachyrium scoparium*^4^	field soil	[glasshouse, 70%]	84	+	0	0	Grman, 2012
19	*Zea mays*^4^	*G. mosseae*	(119, 90, 30.5)^b^	60	+	0	+	Daft and El-Giahmi, 1978
20	*Triticum aestivum*^3^	*Gi. margarita*	325−1025, 72−262	42, 112	+	0	0	Stonor et al., 2014
21	*Flindersia brayleana*^2^	field AM fungi	157, 54	180	+	0	0	Gehring, 2003
22	*Vitis vinifera*	mix of 3 species	1100, 500	111	+	0	−	Schreiner and Pinkerton, 2008
23	*Allium cepa*	*Gi. calospora*	(344, 258, 172, 86)^b^	20, 40, 60, 80, 100	+	Varied^g^	−	Furlan and Fortin, 1977

*Since not all publications provided explicitly calculated MGR values, the MGR responses referred to here as positive (+) or negative (−) means any significant difference between the biomass of the mycorrhizal and non-mycorrhizal plants for a given experimental treatment. The effects of light intensity on MGR and on root length colonized by arbuscular mycorrhizal (AM) fungi (% col.) are shown. “+”, positive effect; “−”, negative effect; “0”, absence of a significant effect; “±”, unimodal response, i.e., a significant peak at the medium light intensity. “G”, Glomus; “C”, Claroideoglomus; “Gi”, Gigaspora. Species names are reported as in the original literature. PPFD, photosynthetic photon flux density*.

1 *legume*,

2 *tree*,

3 *C3 grass*,

4 *C4 grass*.

a *The lowest light treatment omitted because of no AM fungal colonization*.

b *Figures roughly converted from lux or W m^−2^*.

c *The highest P level omitted because of no MGR at any light level*.

d *low P/high P*.

e *Konza soil/Fermi soil*.

f *The effect of light on the MGR not specifically elaborated in the paper*.

g *Effect varied with time. Day 100: Peak at 10 klux*.

Due to the context dependency of AM symbiotic functioning (Hoeksema et al., 2010; Grman et al., 2012), AM symbiosis is not always advantageous for plant biomass production and/or fitness (Johnson et al., 1997, 2015; Janos, 2007). Wide range of **mycorrhizal growth responses** (MGR) ranging from positive to negative have been observed for different plant species along environmental gradients. Despite of the potential negative MGR under certain conditions, AM plants might still have a better fitness than the non-mycorrhizal plants because of their better nutrition (Koide, 2010). Nevertheless, the cases of lower growth as well as total P content of AM plants compared to their non-mycorrhizal controls have also been recorded (e.g., Smith and Smith, 2015).

KEY CONCEPT 5 **Mycorrhizal growth response**

Comparison of (total or shoot) biomass production at a given time point between mycorrhizal (AM) and their respective non-mycorrhizal control (NM) plants, commonly used as a proxy for the net effect of the symbiosis on the plants. Various indices are used, e.g., AM-NM, AM/NM, 100 × (AM-NM)/NM, log(AM/NM).

The dynamics of C and P exchanges between the plants and the fungi in AM symbiosis is sometimes explained as a biological market, in which these sources are reciprocally exchanged, with the preferential allocation to the partner offering the best rate of exchange (Kiers et al., 2011; Werner et al., 2014). Other explanations assume that the volumes of exchanged C and P are operated as surplus resources (Kiers and van der Heijden, 2006; Walder and van der Heijden, 2015) or that they are controlled primarily by the actual needs of symbionts (Landis and Fraser, 2008). From those points of view, plants should not supply the AM fungi with C in situations where the primary limitation of growth and/or reproduction is not mineral nutrition, but the energy availability, such as under severe light deprivation. But the extent to which plant can reduce C flux to the AM fungi is highly questionable. Experimental evidence shows that the AM fungi are not eliminated from roots even under very low light intensities (Schubert et al., 1992). An alternative explanation could then be that the root colonization by AM fungi is maintained as an investment for potentially more favorable future (Landis and Fraser, 2008; Walder and van der Heijden, 2015). It is not even clear whether the decrease of AM fungal colonization of roots under low light is actively driven by the host plant or just a passive consequence of lack of assimilates within the roots. Other question is if the observed decreases of symbiotic benefits under light deprivation should be accounted to the active rule of AM fungi consuming a large fraction of plant C budget or whether they should be attributed to the inability of the AM fungi to collect soil nutrients without sufficient C supply from the plant. Also the rate at which both symbionts react to the change of environmental conditions is virtually unknown.

To gain deeper insights into the dynamics of resource exchange in AM symbiosis, we performed an experiment with plants exposed to long- and short-term shading with different intensities (Konvalinková et al., 2015). Here we discuss our data within a broader context of other studies on AM symbiosis functioning under manipulated light conditions. The purpose of this paper is thus to provide a more complete picture of available knowledge on shading responses of mycorrhizal plants and identify knowledge gaps deserving further attention. To streamline the discussion, we focus specifically on the light/energy deprivation by shading and do not include other studies, where the plants were deprived of their energy resource by defoliation or grazing, inevitably including plant injury and loss of photosynthetic tissues.

## Case study: responses of *Medicago truncatula* to experimental shading

Our previous glasshouse experiment (Konvalinková et al., 2015) compared mycorrhizal (*Rhizophagus irregularis*) and non-mycorrhizal (microbial “mock-inoculum”) barrel medic, *Medicago truncatula*, both with a rhizobial symbiont. The shading was applied either long-term (38 days, starting 14 days after sowing) or short-term (last 6 days of the experiment). Four different levels of shade were included in both variants: 100 (unshaded control), 65, 35, or 10% of the incoming light. The 100% light intensity level corresponded to about 40 klux sunlight (photosynthetic photon flux density being approximately 690 μmol m^−2^ s^−1^). ^13^CO_2_ pulse labeling was carried out on selected treatments 3 days before harvest to follow the allocation of recently fixed C into shoots, roots, and the soil.

**Figure 1:**
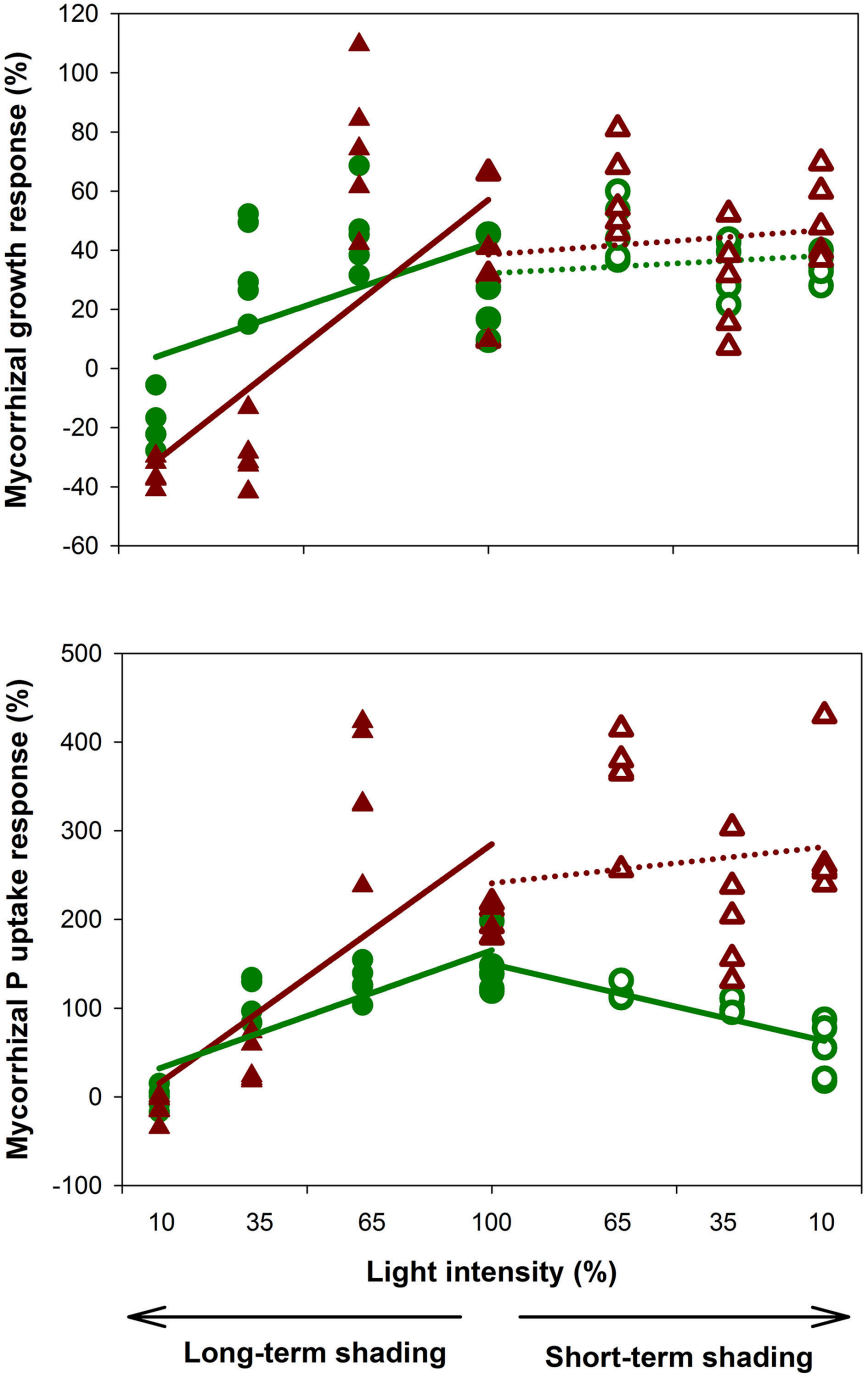
**Growth and phosphorus uptake responses of *Medicago truncatula* plants subjected to experimental shading—either long-term (38 days, closed symbols) or short term (6 days, open symbols) shading, with four different light intensities each**. Five replicate values for shoots (green circles) and roots (brown triangles) per light treatment are shown, together with the linear regression lines testing consistency of the observed effects along the shading gradients (separately for the short- and long-term shading and for the roots and shoots). Mycorrhizal responses were calculated as 100 × (AM-NM)/NM, where AM is the shoot or root dry weight of mycorrhizal plant and NM is the mean value of the respective non-mycorrhizal control treatment (*N* = 5). Solid lines indicate significant trends (*p* < 0.05), whereas dotted lines show lack of statistical significance along the shading gradient.

Experimental plants at full light responded positively to the presence of AM fungus in terms of both biomass production and P content. Upon long-term shading, plant responses to the light gradient were non-linear: while the reduction to 65% of the incoming light intensity caused almost no change to shoot biomass production or shoot P content, there was a marked decrease of these variables under 35 and 10% light intensities. This decrease was more pronounced in AM compared to the non-mycorrhizal plants, resulting in a significant decline of the mycorrhizal growth and P uptake responses along the light intensity gradient applied over a long-term (Figure 1). Hence plants growing at the lowest light intensity showed a clearly negative MGR, whereas the P uptake response at the same light intensity was around zero (Figure 1). Plants adapted to light deprivation over a long-term through reduction of their root-to-shoot biomass ratio and enlargement of the leaflet surface. Interestingly, these morphologic adaptations were further boosted by the presence of mycorrhiza under 35% of incoming light, apparently to compensate for the higher photosynthetic demands of the mycorrhizal plant. Two pieces of evidence indicated a reduction of assimilate flux to the microbial symbionts under long-term shading: First, the mycorrhizal colonization decreased with decreasing light intensity from 71 to 41% of the root length colonized. Second, the isotopic composition of N in plant shoots suggested that AM plants gained higher portion of their N from rhizobia than did the non-mycorrhizal plants at full light, but the situation was reversed at 10% of incoming light intensity—possibly because of relatively higher C supply to AM fungi at the expense of rhizobial symbiont under the C-limited conditions. Our results indicated that mycorrhizal plants were able to compensate for their higher C/energy requirements even when the incoming light intensity dropped to 35% of the ambient light, possibly due their better mineral nutrition. But the compensatory mechanisms failed under the lowest light level, where the mycorrhizal benefits were obviously insufficient to offset the symbiotic costs.

Short-term shading revealed very interesting insights into the dynamics of mycorrhizal functioning. While the levels of mycorrhizal colonization in the roots were unaffected by the different light intensities applied over a short-term, the symbiotic functioning changed rapidly. Plant biomass production decreased with decreasing light intensity independently of the plant mycorrhizal status, confirming the primary limitation of plant growth by light availability over these time scales. Most interestingly, shoot P content of the non-mycorrhizal plants was unaffected by the short-term shading, whereas the shoot P content of the mycorrhizal plants declined rapidly with decreasing light intensity, resulting in gradual decrease of the mycorrhizal shoot P uptake response along the shading gradient (Figure 1). In contrast, the root P content decreased only slightly under the light-shortage for both the mycorrhizal and non-mycorrhizal plant treatments. This resulted in a relative accumulation of P in the roots at the expense of shoots in the shaded mycorrhizal plant. Because normally P is effectively redistributed within the plant body to counteract local P accumulation, P accumulated in roots is supposed to be inside the AM fungal tissues. These results strongly indicate that the AM fungi continued to take up P from the soil despite the light-limitation of their host plant, possibly by mobilizing their internal energy reserves. However, there was a strikingly effective down-regulation of the P transfer from the fungus to the plant, possibly due to decreasing assimilates supply to the roots.

## Overview of arbuscular mycorrhizal functioning under light deprivation

To reveal the importance of light intensity on the functioning of the AM symbiosis, we reviewed previous literature with respect to the outcome of this symbiosis for plant biomass production under varied light conditions (Table 1). We specifically focused on the plant biomass because there were only few studies measuring plant P uptake of mycorrhizal plants exposed to experimental shading. We acknowledge the fact that various ways of calculating the MGR have been used in the literature, or the biomass of AM and non-mycorrhizal plants have been simply compared. For the sake of this review, any significant difference in the above comparisons was reported as a significant MGR. To maintain a strong focus on the potential interaction between the effects of light intensity and AM fungi on the plants, the experiments (or plant-fungal combinations) with no significant effect of at least one of these factors on plant biomass are excluded from Table 1. Similarly, studies with negative impact of high light intensity on the growth of seedling are excluded, because in those studies, the effect of energy-supply along the light-gradient was obviously confounded by other factors (UV, drought stress, etc.). Full list of all reviewed literature is provided as a Supplementary Table to this paper.

We hypothesized that the AM fungi were less beneficial to plant growth under reduced light availability, when the (potential) mycorrhizal benefits were relatively less important as compared to the energy supply. In agreement with our own experimental data (Konvalinková et al., 2015), we found 13 cases of MGR reduced by decreasing light intensity (nos. 1-13 in Table 1). These comprise plants of different functional groups, from usually non-responsive forbs to highly mycorrhiza-responsive legumes and onions, C4 grasses, and seedlings of tropical trees. Nevertheless, there were also 8 cases of MGR independent of light (nos. 15-22 in Table 1) despite the shading levels strong enough to reduce plant growth. Two cases of MGR higher (or rather less negative) under low light (nos. 13-14) and two cases of non-monotonic impact of light on MGR (nos. 12 and 23) were also recorded.

The decrease of MGR with decreasing light intensity can be attributed to the high C demands of the mycorrhizal fungus (nos. 1-3, 5, 9-10, 12). An alternative explanation might be a reduction of direct P-uptake by roots from soil, which is often observed after roots are colonized by AM fungi (Smith et al., 2009), and subsequent P-deficiency of AM plants under the low light, when only low amount of assimilates is available to the fungi. Tester et al. (1985), however, dismissed such an explanation because of non-limiting concentrations of P in tissues of plants growing under low light. This is also confirmed by the fact that shaded AM plants still had higher P concentrations in tissues than the non-mycorrhizal ones, despite the decrease of the MGR (nos. 2, 3, 5-6, 9). Hayman (1974) assumed the C drain to the AM fungi to be too low to cause a decrease of MGR under low light. Unfortunately, studies quantifying the C drain to the AM fungi are still scarce and incoherent. In our experiment (Konvalinková et al., 2015), the positive impact of AM fungi on plant biomass production has turned to negative by a strong light deprivation over a long-term, regardless of the higher P concentrations in tissues of AM- as compared to the non-mycorrhizal plants. This strongly suggests the importance of a mycorrhizal C drain and indicates that C is not operated as a surplus resource under all circumstances. The observed discontinuity of plant growth and mycorrhizal response along the light gradient (Figure 1) might fit with the model of Tuomi et al. (2001), in which plants gain the maximum benefits from the symbiosis when their growth is limited by nutrients (corresponding to our full- and 65% light intensities), but the positive MGR also occurs under C limited conditions, if the AM symbiosis-induced increase of P acquisition allows for the increase in C assimilation high enough to compensate C drain to the fungi (our 35%-light intensity).

Not only plant biomass, but also plant P content was reduced by light-deprivation, and this decrease was stronger for AM- as compared to the non-mycorrhizal plants in our experiment (Konvalinková et al., 2015). Several previous studies also reported that P inflow per unit length of root was reduced by low light intensity and that this decrease was stronger in AM- as compared to the non-mycorrhizal plants (Tester et al., 1985; Son and Smith, 1988; Smith and Gianinazzi-Pearson, 1990). The dependence of mycorrhizal P uptake on light was also illustrated in an experiment with compartmented microcosms, in which unshaded medic plants obtained considerably more radioactively labeled P from AM hyphae than the shaded plants sharing the same microcosm (Fellbaum et al., 2014). Together with the above reviewed decreases in MGR induced by low light intensities, these findings highlight the importance of light supply for the symbiotic functioning of arbuscular mycorrhiza.

Furthermore, because of the tripartite nature (plant-mycorrhiza-rhizobia) of symbiosis in our experimental system and in many other leguminous plant models, the MGR reduction under shade may not be unequivocally and solely explained by reduction of mycorrhizal function. Both the mycorrhizal fungi and rhizobia rely on the same source of C (namely the plant's photosynthesis), and are thus increasingly competing for this resource under C limiting conditions, e.g., under shade. There is at least one more potential feedback between plant nutrition and symbiotic functions, which is related to high P demand of the symbiotic N_2_ fixation (Sulieman et al., 2013). Thus, the decrease of MGR of legumes under shade could also be explained by negative effect of shade on the symbiotic N_2_ fixation—due to lack of P or C or both.

It also needs to be mentioned here that a large functional diversity has been documented among different AM fungal genotypes and species with respect to P acquisition efficiency, as well as C costs incurred (e.g., Munkvold et al., 2004; Lendenmann et al., 2011)—adding further level of uncertainty when comparing different experimental studies considering different AM fungal species and/or communities.

Smith and Read (2008) remark that the negative growth responses are most commonly observed in experiments carried out in glasshouses in winter or in growth rooms with poor light sources. Although meta-analysis of context-dependency of MGR found no significant effect of location (growth chamber or glasshouse vs. field) on MGR in AM symbiosis (Hoeksema et al., 2010), the above mentioned results suggest that the intensity of light might be a crucial factor for the reproducibility of AM research. This might also be one of the reasons of the often found (but not often published) discrepancy between similar experiments carried out at different seasons or in different glasshouses (especially when their cooling systems include shading screens) or growth chambers, the latter often with inherently low light levels as compared to the outdoor conditions.

Another question arises about the relevancy of the tested light conditions in the different experiments for real ecological situations. We previously detected a major impact of light intensity on MGR between 26 and 14 klux (Konvalinková et al., 2015), corresponding to about 450 and 240 μmol m^−2^ s^−1^ photosynthetically active radiation, while the negative impact of AM symbiosis on plant growth was found under 4 klux (~70 μmol m^−2^ s^−1^). These values are not beyond the natural variation of daylight caused by sudden weather changes (at least in Europe), though the last stands for a fairly dark skies (Palz and Greif, 1996). They are also comparable to conditions under a closed tree canopy (Pohlman et al., 2007). Other studies revealing the importance of light supply for MGR operated predominantly with similar intensities (Table 1 nos. 1-6, 10-12, and probably also 9). The studies of Bereau et al. (2000) and Gehring (2003), for example, intentionally simulated light conditions in understory of tropical forest and in the gaps within the closed tree canopy.

Plants have a wide range of mechanisms to adapt to energy-shortage caused by low incoming light intensity as well as by high C demands of the root symbionts. These include changes in morphology, physiology or symbiotic functioning. Enlargement of the photosynthetically active tissues by the presence of AM fungi was observed as an increase in specific leaf area (Wright et al., 1998) or decrease in root-to-shoot or root-to-leaf ratios (Bethlenfalvay and Pacovsky, 1983; Smith and Gianinazzi-Pearson, 1990; Pearson et al., 1991). In our own experiment, we observed the reduction of the root-to-shoot biomass ratio and enlargement of the leaflet surface as a response to AM symbiosis establishment interestingly only under the lower light intensities, suggesting that the assimilate deficiency caused by low light was more pressing in the AM plants (Konvalinková et al., 2015). Similarly, Kyllo et al. (2003) found a significant difference in root-to-shoot ratio between the AM- and non-mycorrhizal tropical shrubs under the low light only, though the difference was only significant for one out of three tested species. To meet their higher C demands, AM plants may also increase the rate of photosynthesis, at least under some conditions (Paul and Kucey, 1981; Wright et al., 1998; Johnson et al., 2015). This is sometimes attributed to the photosynthesis sink strength stimulation (Kaschuk et al., 2009), or thoroughly to the alleviation of sink-limitation of photosynthesis by AM fungi (Louche-Tessandier et al., 1999). However, the last point is unlikely to be broadly valid because the AM plants usually have the same or higher sugar content in leaves than their non-mycorrhizal counterparts (Franken, 2010).

## Plants and fungi—passive pipelines or active players?

Besides others, mycorrhizal plants may also deal with the energy-shortage under low light by reducing assimilate supply to the AM fungi. The decreased extent of fungal colonization of roots under reduced light intensity, indicating activation of such a mechanisms, was observed not only in numerous pot experiments such as those quoted in Table 1, but also in many others (Gamage et al., 2004; Euliss et al., 2007; Olsson et al., 2010; Shi et al., 2014), and in the fields (Heinemeyer et al., 2003; Füzy et al., 2014). Nevertheless, no detectable decrease of fractional AM fungal colonization of roots despite the light limitation of plant growth is also commonly reported from other pot experiments (Table 1). Field studies found no consistent response of AM colonization to the light intensity in the roots of the New Zealand trees (Hurst et al., 2002) or even slightly negative response in the forb *Geranium sylvaticum* (Korhonen et al., 2004), whereas Whitbeck (2001) found positive impact of light intensity on colonization of a tropical tree *Inga leiocalycina* grown in shade houses but not in its natural habitats. The two cases of increased fractional root colonization by shading in the Table 1 were attributed to the vigorous growth of the roots under full light, effectively outgrowing a slower mycorrhizal fungus, which could not have kept the pace with the growth of roots (Furlan and Fortin, 1977; Schreiner and Pinkerton, 2008). In addition, the dependence of C flux to the fungi on the available light is further modulated by the P side of the symbiosis, as revealed by the experiments with combined effects of light and P fertilization: While no effect of light on the root colonization was observed in P-limited soil, the decrease of colonization by the low light occurred after P addition to onion and *Sorghum* (Graham et al., 1982; Son and Smith, 1988; Smith and Gianinazzi-Pearson, 1990) and also in a field experiment on a Tibetan meadow (Liu et al., 2015).

Although the arbuscules (highly branched hyphal structures formed by most AM fungi inside living root cortical cells of their hosts) are expected to be the place of intensive P-for-C exchange between the fungus and the plant (Dickson, 2004; Kiers et al., 2011), the impact of insufficient light on their incidence has only rarely been addressed. For example, Hayman (1974) noticed smaller arbuscules being formed in roots of shaded onions. Similarly, Pearson et al. (1991) reported lower incidence of arbuscules formed by *Glomus mosseae* in roots of leeks plants shaded for up to 1 month, but no longer—consistent with to the absence of long-term (42 days) shading effect on the arbuscules in onions colonized by same fungus (Smith and Gianinazzi-Pearson, 1990). Interestingly, the decrease of arbuscular incidence under shade was found in both pot and field experiments in Tibetan plateau only when fertilizers were added to the soil (Shi et al., 2014; Liu et al., 2015)—indicating a possible interplay between nutritional and energetic status of the host plant playing a role in regulating its mycorrhizal colonization levels.

The question now arises, whether the observed light-induced changes in colonization happen only passively by the low assimilate content of the light-deficient plants or if the plants can actively reduce the C supply to the fungi. This question is challenging because of the fragmentary knowledge of the mechanisms and their regulation of C transport between plant and the AM fungus (Hall and Williams, 2000; Doidy et al., 2012). Furthermore, studies of sugar concentrations in AM roots exposed to the different light conditions are scarce. An earlier study on ash seedlings seems to support the idea of decreased sugar concentration in roots under low light intensities (Borges and Chaney, 1993), but in this case the light was obviously not the primary limitation of plant growth. The study on soybean plants exposed or not to a complete darkness for 12 days showed a decrease in sugar content in the light-deprived plants, going hand in hand with suppressed development of AM fungi in roots of light-deprived plants (Schubert et al., 1992). Interestingly, neither intercellular hyphae nor arbuscules were formed in bean seedlings grown in darkness since their germination, although the hyphal attachments were abundant and appresoria were formed, suggesting the importance of assimilates as signal molecules in AM fungal development (Vierheilig et al., 2002).

Once in the roots, colonization by AM fungi might be reduced by low light, although this is not always the case (see above and in Table 1). Important is that, despite the severe light deprivation levels tested and potential growth depressions in comparison to the non-mycorrhizal plants, the fungi are usually not eliminated from the roots due to light shortage. This indicates that the reduction of AM development in roots is not the common mechanism of compensation of relative increase in the symbiotic costs under persistent light deficiency. In our own experiment, the AM fungi were not eliminated despite the evident plant growth depression. One can argue that plants are actually not able to evaluate the benefits supplied by fungi (Walder and van der Heijden, 2015), for example they have no ability to assess the amount of (scattered and immobile) P, which is available in the soil beyond the root depletion zone (Landis and Fraser, 2008) and, indeed, plant itself has no comparison with the hypothetical non-mycorrhizal state. Thus, the maintenance of the AM fungal colonization in the roots under unfavorable conditions might also be understood as an investment which has not yet returned its benefits, and may or may not return them in the future. However, there is another plausible explanation, relevant for natural settings. It has been long recognized that even plants growing in a deep shade like in a forest understory are often well-colonized by the AM fungi. This can be attributed to the existence of **common mycorrhizal networks (CMN)**, in which shaded plants might gain the benefits from AM fungi, which are actually being fed by the other (neighboring) plants. This has not often been tested, but there is an increasing number of experiments specifically asking this sort of question, using both shaded and unshaded plants interconnected by a shared AM hyphal network (Hodge and Fitter, 2010; Fellbaum et al., 2014; Knegt et al., 2016). Once a common mycorrhizal network has established, one particular plant is unable to impose strong sanctions onto the fungi because the fungus could easily reallocate the resources to the other plant; thus it can only join the network or compete for nutrients with the AM fungi maintained by other plants. This can thus be regarded as a strategy of the AM fungus to maintain the biological markets (Werner et al., 2014).

KEY CONCEPT 6 **Common mycorrhizal network**

Situation where the hyphal network of one mycorrhizal fungal individuum interconnects two to many different plant individuals, belonging to the same or different species. Thus, mycorrhizal benefits and costs for the individual plants can dynamically shift depending on the environmental context, with consequences for plant coexistence and community structure.

Another question is whether the observed decreases in mycorrhizal benefits under low light, namely the decreased P supply to the shoots (Konvalinková et al., 2015), are due to a controlled downregulation of the transfer at the plant-fungal interface or whether this is a result of the fungus running out of its own energy reserves necessary to obtain P from the soil solution. The studies showing preferential allocation of fungal P to the sugar-richer roots (Lekberg et al., 2010; Kiers et al., 2011; Fellbaum et al., 2014) support the active rule of the fungi. But the natural shading events like rainy days often affect large stretches of landscape. What happens when the fungi have no choice of a “better” partner? The short-term shading part of our experiment (Konvalinková et al., 2015) allowed addressing this particular question, at least for the particular plant species under the given conditions. The rapid decline of P uptake to the shoots of AM plants and simultaneous accumulation of P in their roots under the growth-limiting light-deprivation imply accumulation of P in the intraradical hyphae, consistent with earlier observation from monoxenic root cultures (Hammer et al., 2011). These results indicate that the fungi were still able to gain P from the soil (or growth media) despite the energy-limitation of their host, perhaps using their own energy reserves, but they stopped, very rapidly, to supply plants with P. These observations might be important for our understanding of AM symbiotic functioning under changing weather conditions, because they indicate, how active the role is played by the AM fungi in controlling P flux from the soil to the plant.

**Figure 2:**
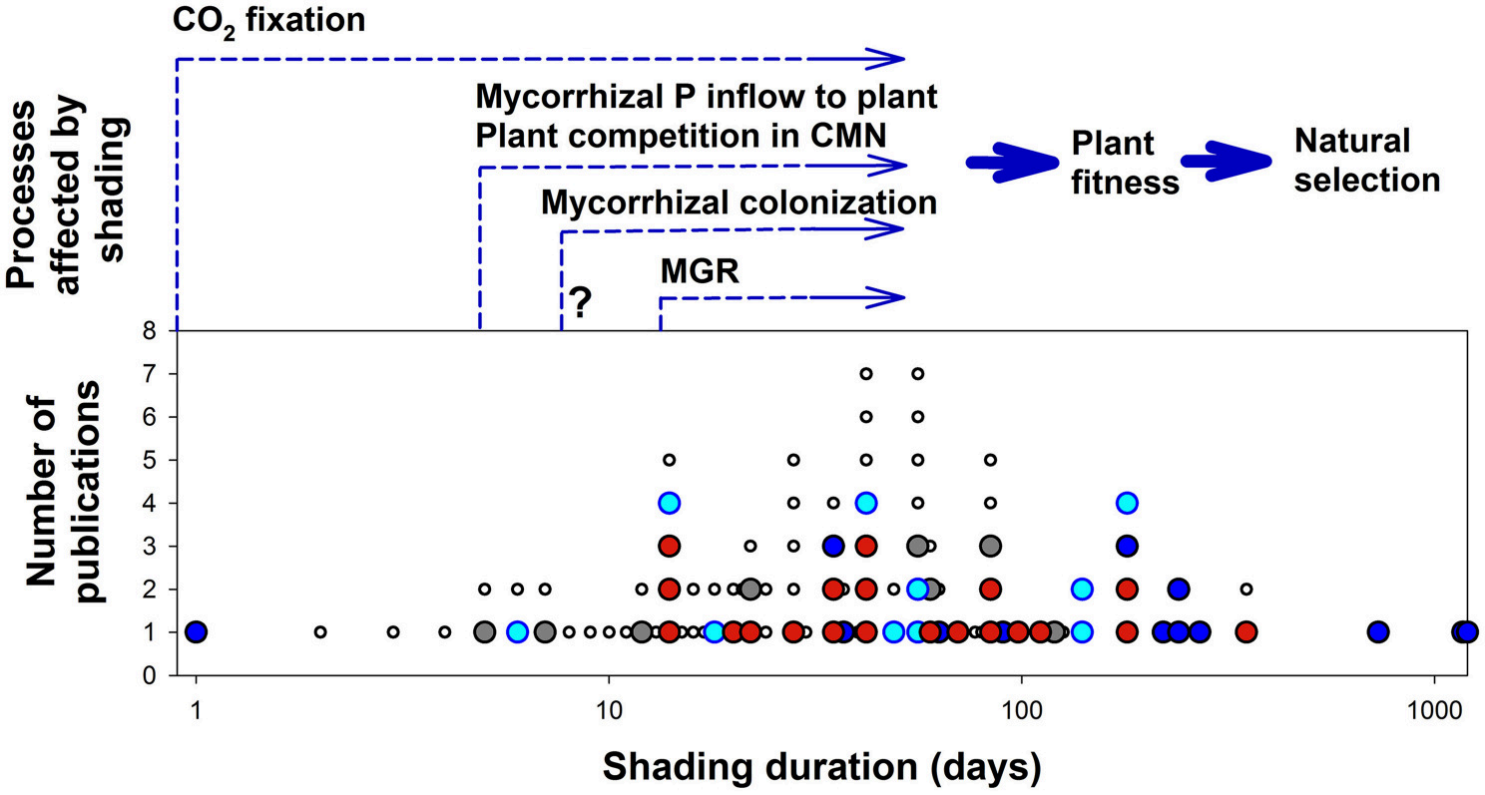
**Plant or plant community processes affected by shading at different temporal scales compared to the lengths of shading period applied in the previously published experiments with arbuscular mycorrhizal plants**. Cumulative numbers of publications are shown for each shading duration. Each closed circle represents one publication, indicating the shortest shading duration tested. Publications shown in red are those listed in Table 1, dark blue are publications without non-mycorrhizal control treatment, light blue are publications with no significant impact of either mycorrhiza or light on the plant biomass, and gray indicates publications with confounding factors such as coincidental treatment with shade and temperature shift or those not reporting on plant biomass or those primarily addressing functioning of common mycorrhizal networks (CMN). Each small empty circle indicates repeated observation (sequential harvest) within a publication that is already shown in the figure. Arrows start at the earliest time point when a shading-induced effect in the respective process has been documented. Please note the log scale of the time axis. P, phosphorus; MGR, mycorrhizal growth response (here regarded broadly as a significant difference in biomass production of mycorrhizal vs. non-mycorrhizal plant at a given time point). Uncertainty related to the onset of the statistically significant difference in mycorrhizal colonization levels between shaded and unshaded plants in the publication by Tester et al. (1986) is indicated with a question mark.

Despite the fact that light conditions often change abruptly under natural settings, the rate at which AM fungi react to these changes remains poorly characterized. So far, we were able to locate only four publications specifically addressing the temporal changes in AM symbiotic functioning due to a sudden change in light conditions over periods shorter than 10 days (Figure 2): Two studies have shown fast decrease of mycorrhiza-mediated P flux to the plants (Fellbaum et al., 2014; Konvalinková et al., 2015), while another found no significant change in mycorrhizal N transport due to shading (Hodge and Fitter, 2010). The remaining study (Saito and Kato, 1994) focused on plants under low light and low temperature stress (simulating cool summer), thus impact of light-shortage alone could not be separated from the other factor here. Obviously, this topic deserves further dedicated attention.

## Conclusions and perspectives

Review of the existing literature on the effects of light intensity on AM symbiosis revealed following aspects of its symbiotic functioning:

- Light/energy deprivation of plants could decrease the MGR, likely due to the symbiotic costs (C transferred to the mycorrhiza) outweighing the symbiotic benefits.
- Plant P uptake is more sensitive indicator of shade-imposed limitation of mycorrhizal functioning than the MGR, yet it has only rarely been measured in the past. Current studies, however, clearly indicated fast downregulation of the fungal P transfer to the plants under light shortage.
- Plants' compensatory mechanisms could buffer the high C costs of the symbiosis over a limited range of light/shade intensities, but they may not be sufficient beyond that range.
- Root colonization levels sometimes react to light-intensity changes, although the active role of plant host in regulating these levels remains unclear.
- Light conditions strongly modulate the outcome of AM symbiosis, ranging from positive to negative effects on the plant host. This appears particularly important for indoor mycorrhizal experiments, where the light provided to the plants should be given special attention.
- It is premature to speculate about mechanisms of regulation of C transfer from the plant to the fungus before the actual (molecular) transfer mechanism is unequivocally demonstrated.

As a particularly important and attainable perspective appears to be a systematic study of the rates of AM fungal and plant responses in terms of their growth and symbiotic exchange of goods such as P and C to experimental shading, covering time scales between hours and several days. Although very relevant from an ecosystem point of view (e.g., cloudy weather), the effect of short-term shading has almost been neglected in past mycorrhizal research. Future efforts should include several (functionally different) plant and fungal genotypes/species, a range of soil properties, and direct isotopic labeling of C, P, and N to account for possible direct and indirect interactions between plant and microbial processes, nutrient availabilities and C allocation. Besides, current high-throughput molecular technologies bear the promise of uncovering the actual molecular transfer mechanisms responsible for these exchanges in a near future (Bravo et al., 2016), opening the door to study the mechanisms of their regulation. A particular attention should be given to the possible changes in turnover of fungal polyphosphates within the AM fungal structures, a process that likely controls the immediate availability to the plant of P transported via mycorrhizal hyphae from the soil to the root cortical cells (Ezawa et al., 2002; Kiers et al., 2011). Ultimately, this research shall allow for better understanding of the magnitude, dynamics, and ecosystem consequences of one of the largest and fastest C flows from plants to soil (i.e., the one mediated by mycorrhizal fungi, Drigo et al., 2010), with particular relevance to soil structure buildup and stabilization of the soil organic matter (Jakobsen and Rosendahl, 1990; Heinemeyer et al., 2006; Verbruggen et al., 2016).

## Author contributions

Both TK and JJ devised the structure and decided on the content of the paper, TK conducted the literature survey, then both TK and JJ jointly wrote the manuscript and contributed to revisions.

## Funding

Authors gratefully acknowledge support by the Czech Science Foundation (project 14-19191S), Ministry of Education, Youth, and Sports of the Czech Republic (project LK11224), and the long-term development program RVO61388971. JJ was also supported by the Fellowship J. E. Purkyně provided by the Czech Academy of Sciences. TK was further supported by the Ministry of Education, Youth, and Sports of the Czech Republic (project LO1417).

### Conflict of interest statement

The authors declare that the research was conducted in the absence of any commercial or financial relationships that could be construed as a potential conflict of interest.
